# Interdomain I/O Optimization in Virtualized Sensor Networks

**DOI:** 10.3390/s18124395

**Published:** 2018-12-12

**Authors:** Congfeng Jiang, Tiantian Fan, Yeliang Qiu, Hongyuan Wu, Jilin Zhang, Neal N. Xiong, Jian Wan

**Affiliations:** 1 Key Laboratory of Complex Systems Modeling and Simulation, Ministry of Education, Hangzhou 310037, China; cjiang@hdu.edu.cn (C.J.); ttfanx@gmail.com (T.F.); qiuyeliang@hdu.edu.cn (Y.Q.); oudongyang1@gmail.com (H.W.); jilin.zhang@hdu.edu.cn (J.Z.); wanjian@zust.edu.cn (J.W.); 2 School of Computer Science and Technology, Hangzhou Dianzi University, Hangzhou 310037, China; 3 College of Intelligence and Computing, Tianjin University, Tianjin 300072, China; 4 Department of Mathematics and Computer Science, Northeastern State University, Tahlequah, OK 74464, USA; 5 School of Information and Electronic Engineering, Zhejiang University of Science and Technology, Hangzhou 310023, China

**Keywords:** interdomain communication, shared memory, circular buffer optimization, virtual sensor networks

## Abstract

In virtualized sensor networks, virtual machines (VMs) share the same hardware for sensing service consolidation and saving power. For those VMs that reside in the same hardware, frequent interdomain data transfers are invoked for data analytics, and sensor collaboration and actuation. Traditional ways of interdomain communications are based on virtual network interfaces of bilateral VMs for data sending and receiving. Since these network communications use TCP/IP (Transmission Control Protocol/Internet Protocol) stacks, they result in lengthy communication paths and frequent kernel interactions, which deteriorate the I/O (Input/Output) performance of involved VMs. In this paper, we propose an optimized interdomain communication approach based on shared memory to improve the interdomain communication performance of multiple VMs residing in the same sensor hardware. In our approach, the sending data are shared in memory pages maintained by the hypervisor, and the data are not transferred through the virtual network interface via a TCP/IP stack. To avoid security trapping, the shared data are mapped in the user space of each VM involved in the communication, therefore reducing tedious system calls and frequent kernel context switches. In implementation, the shared memory is created by a customized shared-device kernel module that has bidirectional event channels between both communicating VMs. For performance optimization, we use state flags in a circular buffer to reduce wait-and-notify operations and system calls during communications. Experimental results show that our proposed approach can provide five times higher throughput and 2.5 times less latency than traditional TCP/IP communication via a virtual network interface.

## 1. Introduction

In a virtualized system, physical resources are abstracted, partitioned, and sliced as virtual resources to virtual machines (VMs). Virtualization provides management convenience and service consolidation, builds a versatile and efficient computing environment to provide services, and achieves an elastic computing architecture and efficient resource utilization. Virtualization essentially enables a system to configure resources from a logical view rather than a physical view. Accordingly, virtualization technology is widely adopted in various online data centers, multitenant cloud-computing platforms, high-performance and -availability clusters, and even high-end desktops [1,2,3].

For sensor networks, virtualization is an important way for provisioning and easy deployment [4,5,6,7,8,9,10,11]. Virtualization in sensor network environments makes it possible for multiple network interface functions to run on a single physical resource for high resource utilization, low cost, and reduced energy consumption for the sensors. In sensor networks, data communication performance has significant impact on both energy consumption and communication latency [12,13,14,15,16,17]. In the emerging edge computing paradigm [18,19], various sensors and edge devices are deployed in smart cities, smart homes, autonomous vehicles, and other smart things. With more powerful computing, storage, and communication capabilities, sensors are no longer constrained to sensing functions, but can provide data storage and even data processing capabilities. These smart sensors are capable of providing complex services with diverse requirements, including data aggregation and analytics. For easier large-scale deployment, manageability, and maintenance, customized/tailored VMs or lightweight containers run atop these smart sensors. Moreover, VMs and containers are easier for sensor programming flexibility, system patching, and updating sensing functions. For example, ad hoc and mission driven sensor networks are highly dynamic due to task reassignment, sensing functions, and mission changes. Virtual machines enable the reprogrammability and flexibility of sensor hardware and mask hardware heterogeneity. Also, processing data after they are sensed at the sensor can yield shorter response times, more efficient processing, and less pressure on the network bandwidth for data aggregation [18,19].

For these smart sensors with VM or container support, interdomain communications are inevitable in many scenarios, including cross-domain data transfer and aggregation, and global data analysis. Since sensors are usually constrained by energy provisioning, highly efficient interdomain communications result in less energy consumption and a longer battery life. However, the scheduler in a traditional VM Monitor (VMM), such as the Xen credit scheduler, is agnostic about the communication behavior between guest operating systems (GuestOS). Experiments in virtualized consolidated environments show that virtualization can significantly increase network communication latency between multiple VMs. For example, CPU (Central Processing Unit) resource allocation methodology has a critical impact on the network latency between collocated VMs when there are CPU and I/O-intensive workloads running simultaneously [20,21,22,23,24,25].

In a virtualized system, I/O virtualization is implemented by abstracting the upper-layer protocols from the physical connections. For network functions, I/O virtualization enables a physical adapter card to appear as multiple virtual network interface cards (vNICs) and virtual host bus adapters (vHBAs), such that vNICs and vHBAs function as conventional NICs and HBAs, and are compatible with existing operating systems, hypervisors, and applications. In networking resources, these virtual network interfaces appear as normal cards, in the same manner as the physical view. In virtualized systems, I/O virtualization simplifies management, lowers operational costs, and improves performance in virtualized environments [26,27,28,29,30,31]. However, I/O virtualization is usually the performance bottleneck in virtualized systems [32,33,34,35].

Virtualization modifies the original hardware, which was deployed on different physical platforms, such that it is integrated into a single physical machine; creating multiple VMs in a single host. The communication between VMs on a single physical machine becomes very complex and frequent. Currently, since communication performance between different VMs on a single physical machine is affected by various aspects, the overhead can largely degrade communication performance [36,37,38]. Memory virtualization is one of the most common virtualization technologies, and the required user memory space may be much larger than the actual size of the machine’s memory. By using memory virtualization technology, part of the hard disk can be virtualized into memory and this is transparent to the user.

Although VM consolidation [39] has seen rapid adoption in practice for easier deployment, the increasing degree of VM consolidation has serious negative effects on VM TCP performance. In a virtualized environment, as multiple VMs share a given CPU, scheduling latencies can substantially deteriorate TCP throughput in the data center [40].

Traditionally, I/O devices are usually exclusively used by a single VM, and thus, become the bottleneck of virtualization performance. To complete interdomain communication between different VMs on the same physical device under the Xen virtualization environment, first a request needs to be sent to the front driver; then, the front driver transfers the request to the corresponding backend driver in Domain0 (the hypervisor domain) [41,42]. In this way, the transferring data first need to be copied from the application to the kernel, then encapsulated by the TCP/IP protocol, and transferred to the other domain through a complex flow control protocol. In addition, multiple check operations, like the checksum and handshake mechanism, need to be done in the transmission process to ensure correct transmission of the information. These result in multiple context switches, and thus degrade communication performance. In addition, the page-flipping mechanism, used to transfer a page between the VMs in the process, needs multiple hypercalls and flushes the page table and TLB (Translation Lookaside Buffer). From the above introduction to the process of the interdomain communication, we can see that traditional interdomain communication can largely degrade communication performance.

This paper presents a model for optimizing interdomain communication between VMs in a single physical machine in a virtualized sensor network environment. This model is based on shared memory and the key idea consists of mapping pages of shared memory directly into the user space, thus getting rid of useless system calls. It can achieve high performance by bypassing the TCP/IP protocol stack and privileged domain, and provides a direct and high-performance communication path between two VMs. The memory is shared via a customized shared device kernel module with a bidirectional event channel residing in both communicating VMs. We use state flags in a circular buffer to reduce wait and notify operations, and thus, system calls. Some evaluations are also presented to show that the optimized model can significantly expand throughput, shorten latency, and improve the CPU utilization of Domain0 compared to the normal interdomain communication method.

The rest of this paper is organized as follows. Section 2 describes related works and background. Section 3 introduces the design and implementation of the optimized model. Section 4 presents the performance evaluation of the optimized model. Finally, we conclude our work in Section 5.

## 2. Related Works

Depending on the different implementations and whether or not the guest operating system kernel code is modified, virtualization technology can be divided into full virtualization, paravirtualization, and hardware-assisted virtualization. Full virtualization depends on a binary instruction translation mode, and no modification is needed to the system kernel code. Paravirtualization needs to modify the guest operating system kernel so that it can efficiently run on a VM manager. Hardware-assisted virtualization, with the help of special hardware instructions, makes the raw operating system run on the VMM. Enterprises use virtualization technology to quickly create services and efficiently manage their business, by achieving more flexible, efficient, and safe management and utilization of different computing resources. Using virtualization technology to create appropriate services in different VMs to achieve service consolidation can make management more convenient, resource utilization more appropriate, and overhead much smaller.

Although Xen provides paravirtualized network architecture, the network performance overhead is significantly heavy. Menon et al. [4] proposed Xenoprof, which is used for network detection and tries to monitor network performance bottlenecks. Xenoprof monitors performance overhead by detecting clock interruptions, cache, block table hits, and other hardware events. In order to improve the Xen network communication performance, Menon et al. [5] also proposed techniques for optimizing network performance in a Xen virtualized environment. First, their technique redefines the virtual network interfaces of guest domains to incorporate high-level network offload features. Second, it optimizes the implementation of the data-transfer path between DomU (the user domain) and Dom0. Last, it provides support for the guest systems to effectively use advanced virtual memory features, such as superpages.

The grant table mechanism of Xen provides an interface to virtual domains to optimize interdomain communication performance with a shared memory page. Inter-VM communication method (IVCOM) [43] applies a direct communication channel between Dom0 and a hardware virtualization-based VM (HV2M) and can greatly reduce VM entry/exit operations, which has improved HV2M performance. Zhang et al. [44] pointed out that the virtual network Xen model has huge overhead due to the inefficiency of interdomain communications and privileged instructions (hypercall), and the network protocol stack. They also propose a fast interdomain communication scheme XenSocket. XenSocket offers a new socket interface. Therefore, one only needs to perform some modifications in the application layer. However, that is a one-way channel, which is different from the traditional view of the socket. XWay [45] provides a two-way channel of communication between domains, which is transparent to part of the upper application. It directly implements a new transparent layer in the INET (Internet) and TCP layers, reduces the processing overhead of TCP/IP and page mapping, and shortens the communication path. However, it only supports TCP communication, and thus, the Linux kernel code needs to be modified. XenLoop [46] demonstrated a shared memory interdomain communication scheme that does not need to modify the kernel, is completely transparent to the upper application, and also supports dynamic migration of VMs. Compared with XWay, which is not completely transparent to the upper application, XenLoop implements interception to the upper message by using the *netfilter* function library, which is fully compatible with upper network applications. Huang et al. [47] proposed IVC (Inter-VM Communication), an interdomain communication scheme that is based on the message passing mechanism allowing a high-performance computer program based on the MPI (Message Passing Interface) library to communicate by shared memory. However, IVC differs from the previous XenSocket and XWay in that it provides migration support and automatic domain discovery but needs to do some modifications in the kernel and the upper application. AdaptIDC [48] is an interdomain communication system that implements an adaptive shared memory. With the help of the IOIHMD (Immediate On-demand Increase and Hysteretic Multiplicative Decrease) adjustment algorithm, the control ring, and the event channel reuse mechanism, AdaptIDC achieves superior shared memory utilization and does not sacrifice high-performance between domains.

These schemes, which are based on Xen’s grant table mechanism, are implemented by the producers–consumers buffer. The user cannot directly access the shared memory space; they can only read and write the shared memory through system calls, which lead to context switches and have an impact on performance. Among these schemes, XenLoop’s performance is the worst, which is partly because of the overhead of the *netfilter* function [49]. XWay, XenSocket, and IVC achieve better network performance by directly placing the data into the shared memory, but, at the same time, they cannot guarantee transparency of the upper application.

Compared to the above interdomain communication mode, based on shared memory [6,50,51,52,53,54], Fido et al. [50] implemented interdomain communication by using full page mapping. Pages are mapped to the other side, so the other side can directly read and write the corresponding page while communicating. Zhang et al. [55] presented MemPipe, a dynamic shared-memory management system for high-performance network I/O among VMs located on the same host. For big data and latency-sensitive applications in virtualized systems, memory is increasingly becoming a bottleneck, and memory efficiency is critical for the high-performance execution of VMs, especially for changing workloads [7,56,57]. Modern complex embedded systems use memory partitioning to satisfy a wide set of nonfunctional requirements, such as strong temporal and spatial isolation [8,58,59]. Oliveira et al. [58] presented TZ-VirtIO, an asynchronous standardized interpartition communication (IPC) mechanism on top of a trust zone-assisted dual-OS hypervisor (LTZVisor) using a standard VirtIO transport layer. Smith et al. [60] proposed a system for dynamically allocating memory amongst virtual machines at runtime, and they evaluated six allocation policies implemented within the system. Zhang et al. [61] proposed iBalloon, a light-weight, accurate and transparent prediction based mechanism to enable more customizable and efficient ballooning policies for rebalancing memory resources among VMs.

In order to maximize the effectiveness of virtualization systems where resources are allocated fairly and efficiently amongst VMs, Smith et al. [30] presented a system for dynamically allocating memory among VMs at runtime. They also provided evaluations of six allocation policies implemented within the system. In their system, they allowed guest VMs to expand and contract according to their changing demands by uniquely improving and integrating mechanisms such as memory ballooning, memory hotplug, and hypervisor paging.

Disk I/O performance is vital for virtualized systems like HPC clusters or commodity servers [12,13,26,29,30,32,33,62,63]. Zeng et al. [62] proposed Raccoon, a network I/O allocation framework for a workload-aware VM scheduling algorithm, to facilitate hybrid I/O workloads in virtual environments. Raccoon combines the strengths of the paravirtualized I/O and SR-IOV techniques to minimize network latency and optimize bandwidth utilization for workload-aware VM scheduling. In the area of high-performance computing, DMA (Direct Memory Access)-capable interconnections provide ultralow latency and high bandwidth in distributed storage and data-processing systems. However, it is difficult to deploy such systems in virtualized data centers due to a lack of flexible and high-performance virtualization solutions for RDMA (Remote Direct Memory Access) network interfaces [64]. Hybrid virtualization (HyV) [28] was proposed to separate paths for control and data operations available in RDMA. In such hybrid virtualization, RDMA control operations are virtualized using hypervisor involvement, while data operations are set up to completely bypass the hypervisor. In order to provide accurate and realtime decision for interdomain communication scheduling, system monitoring is very important for resource utilization in both device level and VM level [65].

Deshpande et al. [34] proposed a traffic-sensitive live VM migration technique to reduce the contention of migration traffic with the VM application traffic. It uses a combination of pre-copy and post-copy techniques for the migration of colocated VMs (those located on the same source host), instead of relying on any single predetermined technique for the migration of all VMs. Memory sharing is used to provide a data transferring venue for data communication between multiple VMs [27,30,32]. Kocoloski et al. [31] present XEMEM, a shared memory system that can construct memory mappings across enclave OSes (Operating Systems) to support composed workloads while allowing diverse application components to execute in strictly isolated enclaves.

Levis et al. [9] presented Maté, a tiny communication-centric VM designed for sensor networks. Maté’s high-level interface allows complex programs to be very short, reducing the energy cost of transmitting new programs, and its code is broken up into small capsules of 24 instructions, which can self-replicate through the network. Packet sending and reception in Maté capsules enable the deployment of ad hoc routing and data aggregation algorithms.

However, even though the solutions above are efficient and very user friendly, they all require system calls (and thus context switching) in order to achieve read-and-write operations, since the user space cannot directly access the shared memory. In this paper, we present an interdomain communication model based on shared memory under a Xen system. This optimized model directly maps the shared page to the user space and reduces unnecessary system calls. Therefore, it substantially increases communication bandwidth and throughput, and effectively improves the communication performance between VMs. The implementation of this optimized model is divided into two parts. The first part is a shared memory device kernel model, which provides a way to share memory between the two domains. The second part is a shared memory interdomain channel interface library. Its main role is to provide a tool that uses the shared memory as the optimized communication channel, and optimizes the ring buffer. The proposed model in this paper is based on shared memory and it can achieve high performance by bypassing TCP/IP protocol stacks and privileged domains, providing a direct and high-performance communication path between two VMs. For easier deployment and flexible management in edge computing environment, an edge device can run in a virtual machine or container. Therefore, the approach proposed in this paper can also be applied in edge computing environment.

## 3. Interdomain I/O Optimization Based on Shared Memory

In this paper, we propose to optimize the interdomain communication between VMs in a single physical machine in a virtualized sensor network environment. Our approach is based on shared memory, and the key technique consists in mapping shared memory pages directly into the user space, thus reducing useless system calls. It can achieve high performance by bypassing TCP/IP protocol stacks and privileged domains, providing a direct and high-performance communication path between two VMs. In our approach, the sending data are shared in the memory and not transferred via a TCP/IP stack. The communication data are directly mapped into the user space of the VM, therefore reducing useless system calls and context switches. Memory is shared via a customized shared device kernel module with a bidirectional event channel residing in both communication VMs. We use state flags in the circular buffer to reduce wait and notify operations, and thus, reduce the system calls.

**Figure 1:**
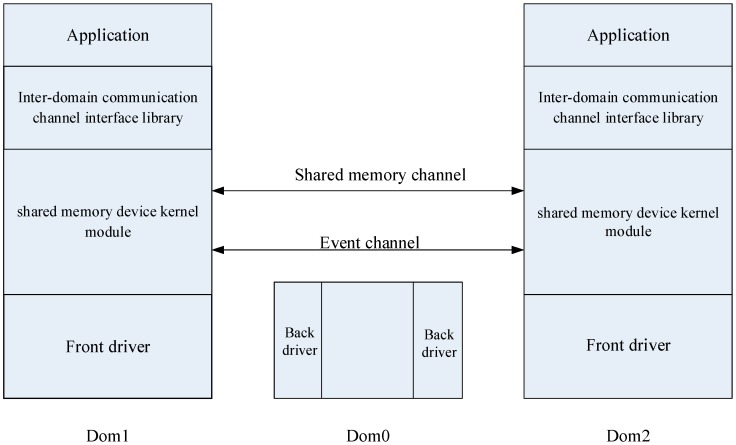
Architecture of the proposed model for interdomain communication optimization.

The overall structure of the optimized model is shown in Figure 1. The model consists of two main parts: the shared memory device kernel module and the interdomain communication channel interface library. Dom0 is the host domain, and Dom1 and Dom2 are two guest VMs colocated on the same hardware.

The shared memory device kernel module is a Linux kernel module that defines a new device driver. It provides a way to share memory between two user spaces of different VMs on the same physical machine. It also uses a bidirectional event channel to provide notifications and responses of messages.

An interdomain communication channel interface library is a user space library that is located on the shared memory device kernel module. It not only provides a file-like interface for users to transfer data using the shared memory device kernel module, but it also implements the optimization of the circular buffer.

In our model, we used header pages to store control information during the memory sharing process, such as the granted privileges index of shared pages, event channel ports, and the communication status of both communicating sides. Once we grant the receiver access privilege to the header pages, and call the hypercall to map the shared memory pages to the receiver’s address space, the receiver can access all the granted pages via the index page. We used the following structure to store the header pages:

*struct xen_shm_header* {

*uint8_t offer_state;*

*uint8_t receiver_state;*

*uint8_t pages_count;*

*evtchn_port_t offer_ec_port;*

*grant_ref_t grant_refs* [*XEN_SHM_ALLOC_ALIGNED_PAGES*]*;*

};

where:

*offer_state* and *receiver_state* stand for the status of the offer and receiver. Here, there are three statuses, i.e., *none*, open, and closed;

*pages_count* stands for the number of shared pages;

*evtchn_port_t* stands for the allocated event channel port number of the offer side, and the port number can be customized in virtualized system;

*grant_refs* stands for the index containing all the granted pages.

We also defined the instance to stand for communication parties, i.e., the offer side provides shared memory pages, while the receiver side maps the shared memory pages into its own address space. Such instance is defined via the structure *xen_shm_instance_data* as the following:

*struct xen_shm_instance_data* {

*uint8_t pages_count;*

*unsigned long shared_memory;*

*domid_t local_domid;*

*domid_t distant_domid;*

*evtchn_port_t local_ec_port;*

*evtchn_port_t dist_ec_port;*

*grant_ref_t first_page_grant;*

*unsigned int offerer_alloc_order;*

struct vm_struct *unmapped_area;

*grant_handle_t grant_map_handles* [*XEN_SHM_ALLOC_PAGES*]*;*

}*;*

where:

*pages_count* is the number of consecutive allocated pages;

*shared_memory* (only in offer side) is the allocated page address;

*local_domid* and *distant_domid* are the local and remote domain IDs, respectively;

*local_ec_port* and *dist_ec_port* are the allocated event channel port number of the local and remote domains, respectively,

*first_page_grant* is the first granted index;

*offerer_alloc_order* (only on offer side) stores order value to calculate the page numbers;

*unmapped_area* (only for the receiver) is the allocated mapping address to receiver;

*grant_map_handles* is the returning value after calling the memory mapping function *HYPERVISOR_grant_table_op* (). This return value is used to terminate memory mapping when communication is terminated.

We describe the architecture proposed in Figure 1 in the following sections.

### 3.1. Shared Memory Device Kernel Module

The shared memory device kernel module in Figure 1 is a Linux kernel module that defines a device driver. This kernel module not only provides a way of sharing memory, but also uses the bidirectional event channel to provide notifications and response of messages.

In a normal Xen platform, each virtual guest operating system has its own memory address space that is mapped to nonoverlapping physical memory, providing ownership of the memory space to each guest operating system. However, a Xen hypervisor can also remap the memory of the guest system to the address space of the other guest system, which is implemented by the grant table mechanism provided by Xen. Each virtual domain has its own grant table, which is a shared data structure in a Xen system. The grant table keeps the shared grant information that is provided to other domains. The grant table is a page-based mechanism, and these pages can be represented by an integer that is called the grant reference. The grant reference points to a certain entry of the grant tables. The process of the grant access mechanism is as follows: First, guest operating system A must request that the hypervisor grants the right of operating system B to use part of the physical memory of A. Then, the hypervisor issues a ticket called a grant reference. B uses this ticket to map the memory of A to its own address space.

In order to implement the shared memory, the memory first needs to be allocated to one of the two guest operating systems, this is called the *offer*. As granting rights and mapping memory are based on one of the guest operating systems, the other side is called the *receiver*.

Memory creation on the receiver’s side is listed in Algorithm 1. Similarly, memory creation on the offer’s side is listed in Algorithm 2.

**Algorithm 1** Mapping memory creation on the data receiver side**Input**: data size **Output**: mapped memory page in receiver side $\begin{array}{l} 1:\;//allocate\;header\;pages\;for\;shared\;memory\\ 2:\;alloc\_vm\_area(PAGE\_SIZE*XEN\_SHM\_ALLOC\_ALIGNED\_PAGES);\\ 3:\;//Set\;the\;GNTMAP\_host\_map\;type\;and\;map\;header\;pages\\ 4:\;gnttab\_set\_map\_op(\&map\_op,addr,GNTMAP\_host\_map,first\_page\_grant,\;dis\tan t\_domid)\\ 5:\;HYPERVISOR\_grant\_table\_op(GNTTABOP\_map\_grant\_ref,\;\&map\_op,\;1)\\ 6:\;//Map\;memory\;pages\\ 7:\;for\;(page=0;\;page<data->pages\_count;\;page++)\;\{\\ 8:\;gnttab\_set\_map\_op(\&map\_op,addr+page*PAGE\_SIZE,GNTMAP\_host\_map,\\ \;first\_page\_grant,\;dis\tan t\_domid);\\ 9:\;HYPERVISOR\_grant\_table\_op(GNTTABOP\_map\_grant\_ref,\;\&map\_op,\;1);\\ 10:\;//reclaim\;memory\;pages\\ 11:\;free\_vm\_area(data->unmapped\_area); \end{array}$

**Algorithm 2** Mapping memory creation on the data offer side**Input**: data size **Output**: mapped memory page in offer side $\begin{array}{l} 1:\;if(!memoryOverflow)\\ 2://allocation\;memory\;head\;page\\ 3:\;head\_alloc\neg \_get\_free\_pages(GFP\_KERNEL)\\ 4://shared\;pages\;memory\;allocation\\ 5:\;shared\_alloc\neg \_get\_free\_pages(GFP\_KERNEL,order)\\ 6:end\;if\\ 7:\;//grant\;access\;privileges\;to\;receiver\\ 8:\;if(!granted)\\ 9:\;gnttab\_grant\_foreign\_access(data->dis\tan t\_domid,virt\_to\_mfn(header\_p),0);)\\ 10:\;//grant\;access\;privileges\;of\;each\;memory\;pages\;to\;receiver\\ 11:for\;(page=0;page<data->pages\_count;page++)\\ 12:\;header\_p->grant\_refs[page]=gnttab\_grant\_foreign\_access(\\ \;data->dis\tan t\_domid\;,\;virt\_to\_mfn(page\_po\mathrm{int}er),\;0);\\ 13://free\;header\;pages\\ 14:free\_pages(data->shared\_memory,\;data->offerer\_alloc\_order) \end{array}$

Once the memory is created, it can be shared among different VMs. The communication flow on the receiver’s side is described in Algorithm 1. On the receiver’s side, the receiver first calls the function *shmpipe_getdomain id* (shmpipe_p pipe, uint32_t* receiver_domain id) to obtain the receiver’s domain ID and send it to the offer. Then, it receives the offer’s domain ID, grant reference, and *page_count.* Finally, the receiver calls the function *shmpipe_connect* (*shmpipe_p pipe, uint8_t page_count, uint32_t offer_domain id, uint32_t grant_ref*) to connect with the offer.

On the offer’s side (Algorithm 2), the offer first receives the receiver’s domain ID, then obtains its own domain ID and grant reference, and starts to share the memory by calling the function *shmpipe_offers* (shmpipe_p pipe, uint8_t page_count, uint32_t receiver_domain id, uint32_t* offer_domain id, uint32_t* grant_ref). Then, it sends the offer domain ID, grant ref, and *page_count* to the receiver. As a back daemon, it waits for the receiver to connect.

#### 3.1.1. Shared Memory Creation

In order to share memory, the offer asks the hypervisor to grant the right to map the memory using the function *gnttab_grant_foreign_access ()*. The hypervisor stores the corresponding pseudo-physical address, the domain IDs of both ends, and the grant reference. With the domain ID of the offer and the grant reference, the receiver can make another hypercall *HYPERVISOR_grant_table_op ()* to map the memory provided by the offer into its own address space. That is the process of sharing memory.

#### 3.1.2. Instance Initialization

When a user opens the shared memory device, hypercalls are not immediately made to map the memory, but private variables are initialized so that the instance can designate an offer or receiver. To configure the virtual domain instance, specific *ioctl* operations need to be used, and these *ioctl* operations return the value of the local domain ID and grant reference. Then, they share the values so that the other process can call the appropriate *ioctl* operations and configure the virtual domain instance on its side.

In the implementation process, the shared memory is composed of multiple pages, each page having its own grant reference. If all grant references are transmitted through XenStore, there is a huge performance overhead. So, in order to reduce the size of sharing information, our model uses the first page as a header page. This page contains all necessary grant references, event channel information, and state of the communication ends. Therefore, as long as the receiver maps the header page to its own address space, it is able to obtain all of the grant references, and obtains all the information of the shared pages.

#### 3.1.3. Mapping Memory into the User Space

As our optimized model tries to limit kernel involvement as much as possible, the optimized model directly maps the shared memory to the user space; therefore, direct read-and-write operations can be done to reduce performance overheads.

On the offer side, memory mapping is not difficult to implement, as the kernel provides a range of interface functions for the device driver. By calling standard device driver functions (such as *open*, *nmap*, *munmap*, *close*), mapping or unmapping memory can be easily realized. In addition, the function *remap_pfn_range*, used to implement simple remapping, makes remapping easy.

However, on the receiver side, memory mapping is not that simple. Xen API (Application Progamming Interface) can be called with the space address and mapping memory and granting access can be implemented. However, it somehow taints the kernel with page errors during unmapping. So, the right way to work is on a lower level with page tables, and correctly invalidate mapping at an early stage of the memory unmapping.

#### 3.1.4. Event Channel

Shared memory is essential for the communication system but making the system more efficient with only shared memory is not enough. When there are no data to read, a reader must wait. Likewise, when there is no more space, the writer must wait as well. On a single operating system, the kernel is usually responsible for waiting and waking in the process. Mutexs is used to synchronize different processes, but when it comes to different kernels using a shared memory, these mechanisms do not work.

Therefore, in addition to the shared memory, this device module also uses the event channel to provide notification and response to the messages. An event channel is a bidirectional pipe used to transmit and handle virtual interrupts, using an asynchronous event notification mechanism to implement the notification transfer from Xen to the domain. Creating an event channel is similar to sharing memory. The offer opens an event channel, identified by the port number, and the receiver connects to the event channel through port number and remote domain ID. When the offer process initializes its own side, the event channel port number is written into the header page, so the receiver can obtain the port number and connect to the event channel.

#### 3.1.5. Sharing Memory Termination

Due to the nonsymmetrical model of memory sharing, some specific operations are still needed before the model is completely cleaned. The offer has the ownership of the original physical memory and then it allocates the memory, maps the memory into the receiver, and tells the location of the memory to Xen. When closing the module, the offer needs to free the allocated memory or it causes huge memory leakages. However, this optimized model uses direct mapping. If the receiver still has active mapping, it can modify the physical memory. If the offer frees the memory with no special check, this memory is likely to be reallocated, but if at this point the receiver has not been closed and still has active mapping, then it modifies the physical memory, and unspecified errors are expected from the resulting non-desired sharing. The Xen API provides a method to detect the amount of active mapping for each grant. Therefore, the device module avoids memory errors by detecting existing active mapping. 

Kernels at both ends maintain a shared state in the header page, including whether the user is using the opened instance. The shared state fields are monitored, and any waiting process receives an EPIPE (broken PIPE) error once the communication state of the other side becomes closed.

### 3.2. Implementation of the Interdomain Communication Channel Interface Library

Two processes can implement efficient data transmission through sharing memory. Our optimized model provides an optimized channel that not only has excellent performance, but also remains an efficient resource. Thus, the interdomain communication channel interface library not only provides a user-friendly interface for the shared memory device mentioned above, but also implements optimization techniques that have better performance.

Here, we use a circular buffer structure, which offers interesting particularity in that it is wait-free as long as it is neither empty nor full to store the data. The circular buffer is a FIFO (First In First Out) ring buffer. There is a read pointer and a write pointer in the ring buffer, and they share their own cursor position. The read pointer points to the read data in the ring buffer, and the write pointer points to the write data. Reading and writing data in the buffer can be achieved by moving the read and write pointers.

The peers of the communication channel are called reader and writer. One pipe, using an instance of shared memory, needs exactly one writer and one reader, but those roles have no relationship with the underlying offer and receiver roles. Offer and receiver refer to the owner of the physical memory. In the initialization of the communication channel, you need to specify the mode (read or write) and conventions (writer offer or writer receiver).

#### 3.2.1. Circular Buffer Optimization

In this optimized model, we used words instead of bytes to copy data. Since the test system used a 64-bit processor, we used 64 aligned buffers to read and write.

When the circular buffer is full, the writer must wait. Similarly, when the circular buffer is empty, the reader also needs to wait. When the data or space become available, one process needs to notify another process using the notify operation. However, the operations of wait and notify both require a system call. Thus, performance can be optimized by reducing the number of their calls.

First, in order to reduce the number of unnecessary notification calls, we used a sleeping flag to indicate if the process was in a waiting state. Process checks the sleeping flag per read or write call and sends a signal if the sleeping flag of the other peer is set. On the other hand, the sleeping flag is set before the process calls the wait *ioctl*, and it is unset after wake-up.

Then, in order to reduce the number of unnecessary wait calls, a process can loop until data or space are available when the process is alone in the machine. Otherwise, the process would not be scheduled by the kernel scheduler if there are a lot of running processes, which would significantly reduce performance. So, we used an active flag to indicate if the process was active. After setting the active flag, the other process loops until data or space are available, instead of calling the wait *ioctl*.

#### 3.2.2. Deadlock Avoidance

Deadlock is a situation that happens when a process is in a waiting state because the source the process requested is held by another waiting process. If a process cannot change its state because the resource requested by it is being used by another waiting process, then the system is said to be in a deadlock.

Putting a process casually into sleep may cause some problems, because the other process may be waiting. So, we used a flag, waiting, to indicate that a process is waiting for data or space. A process sets the flag whenever it starts waiting for data or space, and unsets it at the end. A process is forbidden from sleeping when the other process sets the waiting flag. Because both processes should not be waiting at the same time, a process should continue looping as long as there are no available data or space. A process sets the waiting flag when it starts each loop, and then checks whether the looping condition is true or not. Therefore, the process knows the other process is waiting, which prevents the process from putting itself to sleep and avoids deadlocks.

#### 3.2.3. Channel Closing

We used a closed flag to indicate a process closes the communication channel, so that the other peer knows. After the closed flag is set, a write call fails to work, and a read call returns the end of file as soon as there are no more available data.

If some process crashes, the system kernel closes the device file and modifies the shared state maintained in the header page. By monitoring the shared state, the kernel sends a signal so that any waiting process returns with the EPIPE error.

The flags and mechanisms we used in our paper can implement termination and also avoid deadlocks.

### 3.3. Interdomain Communication Process

We depict the algorithm of interdomain communication of the proposed model in Algorithm 3.

First, the offer allocates memory, including the shared memory pages and the abovementioned header page, which is used to store some essential control information. The header page is mainly used to store all the grant references of the shared memory pages, the state information of the communication ends, and the port number of the corresponding event channel. As long as the receiver maps the header page to its own address space, it can obtain all of the grant references and all the information of the shared pages. Then, the offer calls function *gnttab_grant_foreign_access* () to grant access to the header page. After that, the offer calls hypercall *HYPERVISOR_event_channel_op* () to assign an unbounded event channel, and then binds the related handler function to the event channel.

As for the receiver end, it first allocates a virtual memory address to map the shared memory pages. Then, by calling hypercall *HYPERVISOR_grant_table_op* (), the receiver maps the header page to its own address space and obtains the grant references of the other shared memory pages in the header page. After that, the receiver maps the other shared memory pages on its own address space in the same way. After page mapping, the receiver obtains the port number of the event channel from the header page and binds the corresponding port number. Finally, it binds related handler function to the event channel.

**Algorithm 3** Interdomain communication process**Input**: domain ID **Output**: mapped memory address $\begin{array}{l} 1:if\left(role=receiver\right)\{\\ 2://\mathrm{Allocate}\;\mathrm{the}\;\mathrm{shared}\;\mathrm{memory}\;\mathrm{pages}\;\mathrm{and}\;\mathrm{the}\;\mathrm{header}\;\mathrm{page}\\ 3://get\;receiver’s\;DomID\;to\;send\;it\;to\;offer;\\ 4:\;shmpipe\_getdomain\;id(shmpipe\_p\;pipe,\;uint32\_t*\;receiver\_domain\;id)\\ 5://Receive\;offer’s\;domain\;id,\;Grant\;ref\;and\;page\_count\;to\;offer\;in\;grant\;table;\\ 6://connect\;with\;the\;offer;\\ 7:\;shmpipe\_connect(shmpipe\_p\;pipe,\;uint8\_t\;page\_count,\;uint32\_t\;offer\_domain\;id,\;uint32\_t\;grant\_ref)\\ 8://map\;the\;memory\;into\;receiver’s\;address\;space\\ 9:\;HYPERVISOR\_grant\_table\_op();\\ 10:\}\\ 11:elseif\left(role=offer\right)\{\\ 12://get\;mapping\;right\\ 13:\;gnttab\_grant\_foreign\_access()\;;\\ 14://Receive\;receiver’s\;domain\;id\\ 15://Get\;offer’s\;domain\;id\;and\;grant\;ref\\ 16://share\;the\;memory\\ 17:\;shmpipe\_offers(shmpipe\_p\;pipe,\;uint8\_t\;page\_count,\;uint32\_t\;receiver\_domain\;id,\;uint32\_t*\;offer\_domain\;id,\;uint32\_t*\;grant\_ref)to\;;\\ 18://Send\;offer\;domain\;id,\;grant\;ref\;andpage\_count\;to\;the\;receiver;\\ 19://Wait\;for\;the\;receiver\;to\;connect;\\ 20:\;wait();\\ 21:\} \end{array}\}$

## 4. Experiment Results

Our evaluations aimed to compare the communication performance of the optimized model with the traditional TCP/IP mode. The evaluations verified that our optimized model has better communication performance than the traditional TCP/IP mode in tests of throughput, communication delay, CPU utilization, and number of hypercalls.

Evaluations used an Intel Core i5-2400 4-core processor, CPU frequency was 3.1 GHz, memory capacity was 4 GB, and we installed Xen 4.2.1. All physical machines and VMs were running CentOS 6.2 with Kernel 3.5.7.

Dom0 and DomU were installed with CentOS Linux, Dom0 was configured with two CPU cores and 2 GB RAM, and each of the two DomUs were configured to use a virtual CPU (VCPU) and 1 GB memory. The testing environment configuration is shown in Table 1.

All interdomain communication experiments were conducted ten times, and the results shown in the following sections are the average values of the ten runs.

### 4.1. Throughput of Interdomain Communications

**Figure 2:**
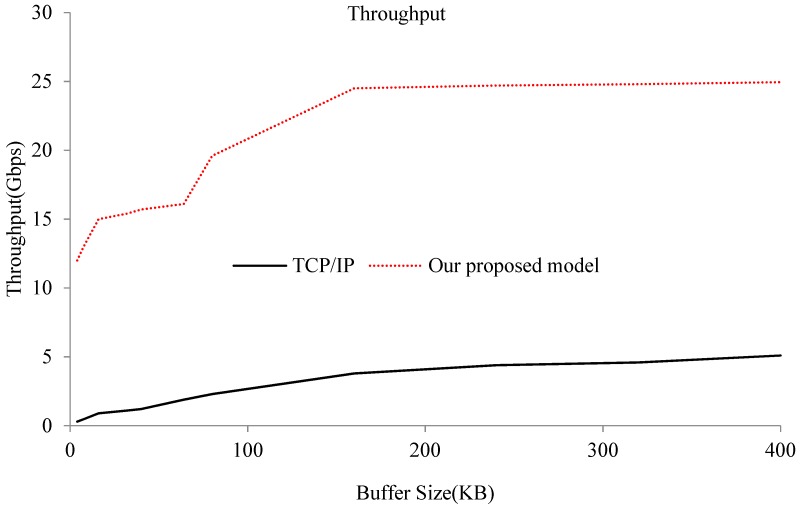
Throughput of the TCP/IP-based (black-solid line) and our optimized (red-dashed line) interdomain communication models.

Throughput capacity is an important performance index for measuring the communication between different guest VMs on the same physical machine. In this evaluation, we first used *iperf* to test throughput capacity between the different guest virtual domains under the original Xen network virtualization infrastructure, TCP/IP. We implemented a test function that could change the buffer size, and one process sends a bunk of messages to a remote domain where another process waits to receive data. Since a TCP/IP-based approach is the main solution for interdomain communication among multiple VMs, we tested the throughput capacity of the optimized model according to this test program compared with the TCP/IP-based performance. We changed the buffer size to investigate the performance limit of our proposed approach. The test results are shown in Figure 2.

Figure 2 shows the throughput comparison of the optimized model and traditional TCP/IP communications. The experimental results show that, compared to the traditional TCP/IP communication, our proposed optimized model had much higher throughput capacity for different buffer sizes. Our optimized approach improves the throughput capacity up to 24 Gbps (the upper limit of the data-access performance of underlying memory modules), which is increased by about five times compared to a traditional TCP/IP-based solution. This means that our optimized approach can exploit the hardware capacity of the underlying memory modules for interdomain communications and it has better communication performance for use in applications that require frequent communication. When buffer size increases, the throughput of both the TCP/IP-based solution and our proposed approach increase accordingly. However, our proposed approach always outperforms the TCP/IP-based solution.

### 4.2. Latency of Interdomain Communications

**Figure 3:**
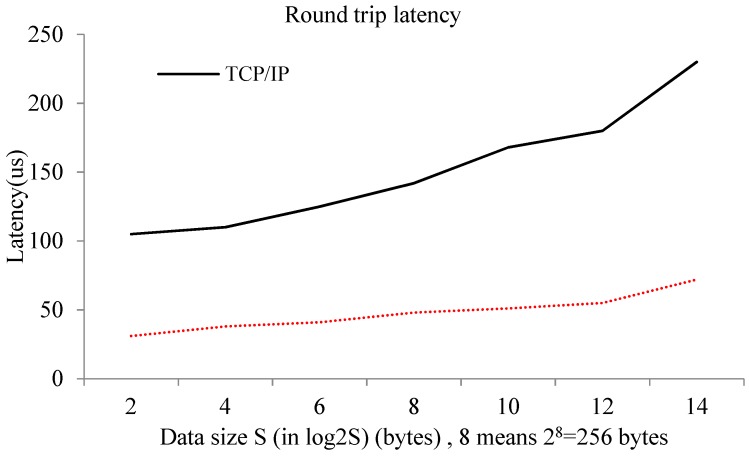
Round-trip latency at different transferring data sizes.

Another important performance indicator of the communication between different guest VMs on the same physical machine is delay. In order to prove that the optimized model is more efficient than a traditional TCP/IP communication approach, the round-trip delay when two VMs send different sized messages to each other (taking into account the impact of bandwidth, the value of the transmission message size is much smaller) needs to be tested. Each experiment was implemented 100 times, and then the average round-trip latency over all experiments was determined. The experimental results are shown in Figure 3.

The experimental results show that, compared to a traditional TCP/IP mode, the optimized model has higher performance. The average delay of the optimized system was 43 µs, while the average delay of the TCP/IP communication mode was 151 µs. The round-trip delay of the optimized model was reduced by approximately four times. We can also see from the figure that with the growth of the size of the transmitted message, both the delay of the TCP/IP and the optimized model increased, and the performances thus dropped. However, the performance decline of the optimized model was not as prominent as that of the TCP/IP mode. This means that the size of the message had smaller impact on the optimization model. The experiments show that the optimized model would be suitable for applications that have limitations on round-trip delays of system communication. In summary, our proposed shared memory-based approach can achieve much higher memory access bandwidth for interdomain communications because it bypasses the lengthy communication path of TCP/IP communications. Our performance is only restricted by the memory bandwidth of the underlying DRAM, whose data-access bandwidth is much higher than that of the underlying NIC in the traditional TCP/IP-based approach. Moreover, in a virtualized environment, NIC is prone to be a bottleneck for packet processing due to the data contention of multiple colocated VMs.

### 4.3. CPU Utilization of Colocated Domains

**Figure 4:**
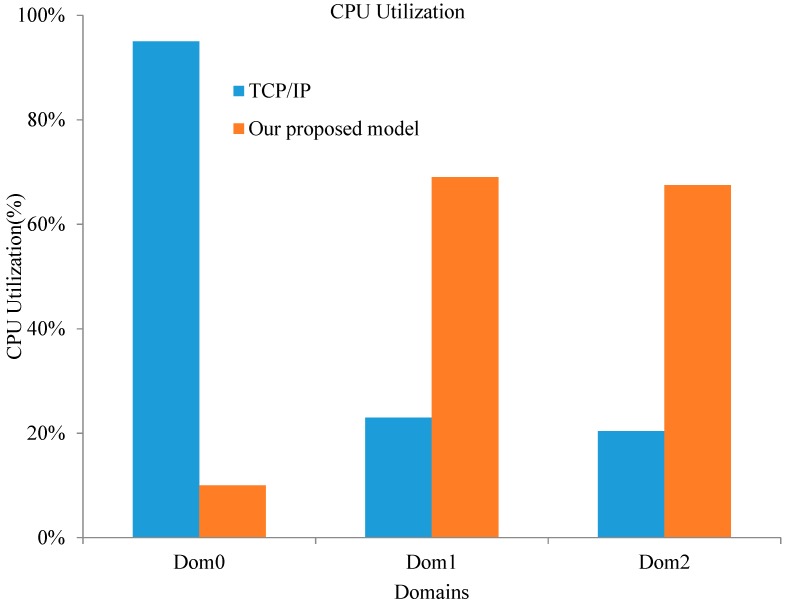
CPU utilization of different virtual machine (VM) domains.

In order to prove that our optimized model is more efficient than the normal TCP/IP mode, evaluations were also performed to test the CPU utilization of the domains. When data were transferred, we collected the CPU utilization of Dom0 and DomU by using the xentop command. The command is *xentop -b 1 -i 200 | awk* ‘*BEGIN* {*print* “*name cpu%*”}{ *print $1*“ ”*$4*}’. This command evaluation sets the update time to 1 s and the number of iterations to 200, then it prints the collected CPU utilization data through *awk*. Finally, we calculated the average CPU utilization of each virtual domain. The experimental results are shown in Figure 4.

Figure 4 demonstrates the comparison of the optimized model and the traditional TCP/IP communication. Evaluation results show that the optimized model significantly reduces CPU utilization in Dom0; the average CPU utilization in normal TCP/IP mode was 95%, and the average in the optimized model was 10%. The reduction of CPU utilization in Dom0 means that Dom0 can schedule and manage more VMs to achieve an efficient use of server and resource integration. Compared to a substantial reduction of CPU utilization in Dom0, CPU utilization in Dom1 and Dom2 increased to a great degree, from 22 to 69%. This is because the optimized model is based on shared memory, and data are transferred through shared memory to improve system throughput and reduce I/O wait time. Therefore, the processing speed of data increases and results in the growth of CPU utilization.

### 4.4. Hypercalls and Context Switches during Interdomain Communications

Another important performance indicator of the communication between different guest VMs on the same physical machine is the number of hypercalls and context switches. In the evaluation, we set the buffer size to 32 KB; then, VM1 sent a 1 GB message to VM2.

We used *perfc* to evaluate the number of hypercalls and context switches. *Perfc* is a building-time option of the Xen hypervisor; we could set *perfc* = *y* to use the *perfc* tool. This is a performance testing tool that tests Xen in a microcosmic way. By using the command *xm debug-keys p*, we could set a *perf* counter and print the results. The evaluation results are shown in Table 2.

As can be seen from Table 2, compared to the TCP/IP mode, the optimization model has better performance. The number of hypercalls was reduced from 108,546,722 to 3,434,825, which is an improvement of roughly 31.6 times. The number of context switches was reduced from 97,580,861 to 86,340, which was a reduction of nearly 1130.2 times. This is because data transmission was complemented by interdomain shared memory in the optimized model, bypassing the device-driver domain, and significantly reducing the number of hypercalls and context switches in the data-transfer process.

## 5. Conclusions

Currently in virtualization environments, communication between different VMs on the same physical machine becomes more frequent. However, the performance of the communication between different VMs on the same physical machine is affected in many aspects that have caused great performance overhead and resulted in reduced communication performance.

This paper presents an interdomain communication model based on shared memory under the Xen system. This optimized model directly maps a shared page to the user space, reduces unnecessary system calls, substantially increases communication bandwidth and throughput, and effectively improves the communication performance between VMs.

The evaluation results show that this optimized model significantly improves throughput between different virtual domains, reduces communication delay, and has better communication performance. However, this model does not guarantee communication security mechanisms and does not take into account the safety of the shared memory, assuming that both ends are trusted domains, like in a private cloud platform environment. Thus, the next step is to obtain a number of mechanisms to improve security.

## Figures and Tables

**Table 1:** Testing environment configuration.

Domain	CPU	Memory
Domain0	Intel Core i5-2400 3.1 GHz	4 GB
DomainU	1 VCPU	1 GB

**Table 2:** Number of hypercalls and context switches.

Perfc Event	TCP/IP-Based	Our Model	Improvement
Hypercalls	108,546,722	3,434,825	31.6 times better
Context switches	97,580,861	86,340	1130.2 times better

## References

[1] García-Valls M., Cucinotta T., Lu C. (2014). Challenges in real-time virtualization and predictable cloud computing. J. Syst. Arch..

[2] Silakov D.V. (2012). The use of hardware virtualization in the context of information security. Program. Comput. Softw..

[3] Jiang C., Wang Y., Ou D., Li Y., Zhang J., Wan J., Luo B., Shi W. (2017). Energy efficiency comparison of hypervisors. Sustain. Comput. Inform. Syst..

[4] Menon A., Santos J.R., Turner Y., Janakiraman G.J., Zwaenepoel W. Diagnosing performance overheads in the Xen VM environment. Proceedings of the ACM SIGOPS/SIGPLAN International Conference on Virtual Execution Environments (VEE).

[5] Menon A., Cox A.L., Zwaenepoel W. Optimizing network virtualization in Xen. Proceedings of the USENIX Annual Technical Conference (ATC).

[6] Mann Z.A. (2018). Resource Optimization across the Cloud Stack. IEEE Trans. Parallel Distrib. Syst..

[7] Xu C., Wang H., Shea R., Wang F., Liu J. (2017). On Multiple Virtual NICs in Cloud Computing: Performance Bottleneck and Enhancement. IEEE Syst. J..

[8] Pérez H., Gutiérrez J. (2016). Enabling Data-Centric Distribution Technology for Partitioned Embedded Systems. IEEE Trans. Parallel Distrib. Syst..

[9] Levis P., Culler D. Maté: A tiny VM for sensor networks. Proceedings of the 10th International Conference on Architectural Support for Programming Languages and Operating Systems.

[10] Reijers N., Ellul J., Shih C. (2018). Making sensor node VMs work for real-world applications. IEEE Embed. Syst. Lett..

[11] Delgado C., Canales M., Ortín J., Gállego J.R., Redondi A., Bousnina S., Cesana M. (2018). Joint Application Admission Control and Network Slicing in Virtual Sensor Networks. IEEE Internet Things J..

[12] Nkomo M., Hancke G.P., Abu-Mahfouz A.M., Sinha S., Onumanyi A.J. (2018). Overlay Virtualized Wireless Sensor Networks for Application in Industrial Internet of Things: A Review. Sensors.

[13] Leee C., Strazdins P. An Energy-Efficient Asymmetric Multi-Processor for HPC Virtualization. Proceedings of the 2018 IEEE International Parallel and Distributed Processing Symposium Workshops (IPDPSW).

[14] Shao C., Tanaka S., Nakayama T., Hata Y., Muroyama M. (2018). Electrical Design and Evaluation of Asynchronous Serial Bus Communication Network of 48 Sensor Platform LSIs with Single-Ended I/O for Integrated MEMS-LSI Sensors. Sensors.

[15] Park S., Kim C.H., Rhee J., Won J., Han T., Xu D. (2018). CAFE: A Virtualization-Based Approach to Protecting Sensitive Cloud Application Logic Confidentiality. IEEE Trans. Dependable Secur. Comput..

[16] Rauniyar A., Engelstad P., Østerbø O.N. (2018). RF Energy Harvesting and Information Transmission Based on NOMA for Wireless Powered IoT Relay Systems. Sensors.

[17] Moon J., Jung I.Y., Yoo J. (2017). Security Enhancement of Wireless Sensor Networks Using Signal Intervals. Sensors.

[18] Shi W., Cao J., Zhang Q., Li Y., Xu L. (2016). Edge Computing: Vision and Challenges. IEEE Internet Things J..

[19] Taherizadeh S., Jones A.C., Taylor I., Zhao Z., Stankovski V. (2018). Monitoring self-adaptive applications within edge computing frameworks: A state-of-the-art review. J. Syst. Softw..

[20] Gamage S., Kompella R.R., Xu D., Kangarlou A. (2013). Protocol Responsibility Offloading to Improve TCP Throughput in Virtualized Environments. ACM Trans. Comput. Syst..

[21] Guan B., Wu J., Wang Y., Khan S. (2014). CIVSched: A communication-aware inter-VM scheduling technique for decreased network latency between co-located VMs. IEEE Trans. Cloud Comput..

[22] Oballe-Peinado O., Vidal-Verdú F., Sánchez-Durán J.A., Castellanos-Ramos J., Hidalgo-López J.A. (2015). Smart Capture Modules for Direct Sensor-to-FPGA Interfaces. Sensors.

[23] Xu C., Ma X., Shea R., Wang H., Liu J. MemNet: Enhancing Throughput and Energy Efficiency for Hybrid Workloads via Para-virtualized Memory Sharing. Proceedings of the 2016 IEEE 9th International Conference on Cloud Computing.

[24] Wu S., Zhou L., Sun H., Jin H., Shi X. (2016). Poris: A Scheduler for Parallel Soft Real-Time Applications in Virtualized Environments. IEEE Trans. Parallel Distrib. Syst..

[25] Min D., Lee S., Byeon G., Hong J. Delay-based scheduling to enhance fairness in a VM environment. Proceedings of the 31st Annual ACM Symposium on Applied Computing.

[26] Zhang J., Lu X., Arnold M., Panda D.K. MVAPICH2 over OpenStack with SR-IOV: An Efficient Approach to Build HPC Clouds. Proceedings of the 2015 15th IEEE/ACM International Symposium on Cluster, Cloud and Grid Computing.

[27] Li S., Zhang Y., Hoefler T. (2018). Cache-oblivious MPI all-to-all communications based on Morton order. IEEE Trans. Parallel Distrib. Syst..

[28] Pfefferle J., Stuedi P., Trivedi A., Metzler B. A hybrid I/O virtualization framework for RDMA-capable network interfaces. Proceedings of the 11th ACM SIGPLAN/SIGOPS International Conference on Virtual Execution Environments.

[29] Li D., Jin H., Liao X., Zhang Y., Zhou B. (2013). Improving disk I/O performance in a virtualized system. J. Comput. Syst. Sci..

[30] Li D., Dong M., Tang Y., Ota K. (2018). A novel disk I/O scheduling framework of virtualized storage system. Clust. Comput..

[31] Kocoloski B., Lange J. XEMEM: Efficient Shared Memory for Composed Applications on Multi-OS/R Exascale Systems. Proceedings of the 24th International Symposium on High-Performance Parallel and Distributed Computing.

[32] Zhou Z., Yu M., Gligor V.D. Dancing with Giants: Wimpy Kernels for On-Demand Isolated I/O. Proceedings of the 2014 IEEE Symposium on Security and Privacy.

[33] Zhang J., Lu X., Panda D.K. High-Performance VM Migration Framework for MPI Applications on SR-IOV Enabled InfiniBand Clusters. Proceedings of the 2017 IEEE International Parallel and Distributed Processing Symposium (IPDPS).

[34] Deshpande U., Keahey K. (2017). Traffic-sensitive live migration of VMs. Future Gener. Comput. Syst..

[35] Xi S., Li C., Lu C., Gill C. Prioritizing local interdomain communication in Xen. Proceedings of the 2013 IEEE/ACM 21st International Symposium on Quality of Service (IWQoS).

[36] Ram K.K., Santos J.R., Turner Y. (2010). Redesigning Xen’s Memory Sharing Mechanism for Safe and Efficient IO Virtualization. Proceedings of the International Workshop on I/O Virtualization.

[37] Nanos A., Koziris N. (2014). Xen2MX: High-performance communication in virtualized environments. J. Syst. Softw..

[38] Ren Y., Liu L., Zhang Q., Wu Q., Yu J., Kong J., Guan J., Dai H. Residency-Aware VM Communication Optimization Design Choices and Techniques. Proceedings of the 2013 IEEE Sixth International Conference on Cloud Computing.

[39] Han G., Que W., Jia G., Shu L. (2016). An efficient virtual machine consolidation scheme for multimedia cloud computing. Sensors.

[40] Jiang C., Duan L., Liu C., Wan J., Zhou L. (2013). VRAA: virtualized resource auction and allocation based on incentive and penalty. Clust. Comput..

[41] Fremal S., Manneback P. Optimizing Xen inter-domain data transfer. Proceedings of the 2014 International Conference on High Performance Computing & Simulation (HPCS).

[42] Li J., Xue S., Zhang W., Ma R., Qi Z., Guan H. (2018). When I/O Interrupt Becomes System Bottleneck: Efficiency and Scalability Enhancement for SR-IOV Network Virtualization. IEEE Trans. Cloud Comput..

[43] Bai Y., Ma Y., Luo C., Lv D., Peng Y. (2013). A high performance inter-domain communication approach for VMs. J. Syst. Softw..

[44] Zhang X., McIntosh S., Rohatgi P., Griffin J.L. XenSocket: A high-throughput interdomain transport for VMs. Proceedings of the ACM/IFIP/USENIX 2007 International Conference on Middleware.

[45] Kim K., Kim C., Jung S.I., Shin H.S., Kim J.S. Inter-domain Socket Communications Supporting High Performance and Full Binary Compatibility on Xen. Proceedings of the Fourth ACM SIGPLAN/SIGOPS International Conference on Virtual Execution Environments.

[46] Wang J., Wright K.L., Gopalan K. XenLoop: A Transparent High Performance Inter-VM Network Loopback. Proceedings of the 17th International Symposium on High Performance Distributed Computing (HPDC).

[47] Huang W., Koop M.J., Gao Q., Panda D.K. VM aware communication libraries for high performance computing. Proceedings of the 2007 ACM/IEEE Conference on Supercomputing.

[48] Liao X., Chen K., Jin H. (2013). AdaptIDC: Adaptive inter-domain communication in virtualized environments. Comput. Electr. Eng..

[49] Ren Y., Liu L., Liu X., Kong J., Dai H., Wu Q., Li Y. A fast and transparent communication protocol for co-resident VMs. Proceedings of the 8th International Conference on Collaborative Computing: Networking, Applications and Worksharing (CollaborateCom).

[50] Burtsev A., Srinivasan K., Radhakrishnan P., Voruganti K., Goodson G.R. Fido: Fast Inter-Virtual-Machine Communication for Enterprise Appliances. Proceedings of the 2009 USENIX Annual Technical Conference (ATC).

[51] Ning F., Weng C., Luo Y. Virtualization I/O Optimization Based on Shared Memory. Proceedings of the 2013 IEEE International Conference on Big Data.

[52] Jiang C., Wan J., Wu H., Zhang W., Zhang J., Ren Z., Ye Z. Optimized Inter-domain Communications Among Multiple VMs Based on Shared Memory. Proceedings of the 2015 IEEE 17th International Conference on High Performance Computing and Communications (HPCC).

[53] Ren Y., Liu L., Zhang Q., Wu Q., Guan J., Kong J., Dai H., Shao L. (2016). Shared-Memory Optimizations for Inter-Virtual-Machine Communication. ACM Comput. Surv..

[54] Zhang Q., Liu L. Shared Memory Optimization in Virtualized Cloud. Proceedings of the 2015 IEEE 8th International Conference on Cloud Computing.

[55] Zhang Q., Liu L. (2016). Workload Adaptive Shared Memory Management for High Performance Network I/O in Virtualized Cloud. IEEE Trans. Comput..

[56] Zeng L., Wang Y., Kent K.B., Xiao Z. (2017). Naplus: A software distributed shared memory for virtual clusters in the cloud. Softw. Pract. Exp..

[57] Zhang Q., Liu L., Pu C., Cao W., Sahin S. Efficient Shared Memory Orchestration towards Demand Driven Memory Slicing. Proceedings of the 2018 IEEE 38th International Conference on Distributed Computing Systems.

[58] Oliveira A., Martins J., Cabral J., Tavares A., Pinto S. TZ- VirtIO: Enabling Standardized Inter-Partition Communication in a Trustzone-Assisted Hypervisor. Proceedings of the 2018 IEEE 27th International Symposium on Industrial Electronics (ISIE).

[59] Garcia P., Gomes T., Monteiro J., Tavares A., Ekpanyapong M. (2016). On-Chip Message Passing Sub-System for Embedded Inter-Domain Communication. IEEE Comput. Arch. Lett..

[60] Smith R., Rixner S. A policy-based system for dynamic scaling of VM memory reservations. Proceedings of the 2017 Symposium on Cloud Computing.

[61] Zhang Q., Liu L., Ren J., Su G., Iyengar A. iBalloon: Efficient VM Memory Balancing as a Service. Proceedings of the 2016 IEEE International Conference on Web Services (ICWS).

[62] Zeng L., Wang Y., Fan X., Xu C. (2017). Raccoon: A Novel Network I/O Allocation Framework for Workload-Aware VM Scheduling in Virtual Environments. IEEE Trans. Parallel Distrib. Syst..

[63] Zhang Q., Liu L., Ren Y., Lee K., Tang Y., Zhao X., Zhou Y. Residency Aware Inter-VM Communication in Virtualized Cloud: Performance Measurement and Analysis. Proceedings of the 2013 IEEE Sixth International Conference on Cloud Computing.

[64] Mouzakitis A., Pinto C., Nikolaev N., Rigo A., Raho D., Aronis B., Marazakis M. Lightweight and Generic RDMA Engine Para-Virtualization for the KVM Hypervisor. Proceedings of the 2017 International Conference on High Performance Computing & Simulation (HPCS).

[65] Jiang C., Wan J., Xu X., Zhang J., You X. (2013). Resource Allocation in Contending Virtualized Environments through Stochastic Virtual Machine Performance Modeling and Feedback. J. Inf. Sci. Eng..

